# P53 and the defenses against genome instability caused by transposons and repetitive elements

**DOI:** 10.1002/bies.201600031

**Published:** 2016-05-13

**Authors:** Arnold J. Levine, David T. Ting, Benjamin D. Greenbaum

**Affiliations:** ^1^ Institute for Advanced StudySchool of Natural SciencesThe Simons Center for Systems BiologyPrincetonNJUSA; ^2^ Rutgers Cancer Institute of New JerseyNew BrunswickNJUSA; ^3^ Massachusetts General Hospital Cancer CenterHarvard Medical SchoolCharlestownMAUSA; ^4^ Department of MedicineMassachusetts General HospitalBostonMAUSA; ^5^ Department of Medicine, Hematology, and Medical Oncology, and Pathology, Tisch Cancer InstituteIcahn School of Medicine at Mount SinaiNew YorkNYUSA

**Keywords:** cancer biology, cancer immunology, endogenous elements, genome stability, innate immunity, repetitive elements, transposons

## Abstract

The recent publication by Wylie et al. is reviewed, demonstrating that the p53 protein regulates the movement of transposons. While this work presents genetic evidence for a piRNA‐mediated p53 interaction with transposons in *Drosophila* and zebrafish, it is herein placed in the context of a decade or so of additional work that demonstrated a role for p53 in regulating transposons and other repetitive elements. The line of thought in those studies began with the observation that transposons damage DNA and p53 regulates DNA damage. The presence of transposon movement can increase the rate of evolution in the germ line and alter genes involved in signal transduction pathways. Transposition can also play an important role in cancers where the p53 gene function is often mutated. This is particularly interesting as recent work has shown that de‐repression of repetitive elements in cancer has important consequences for the immune system and tumor microenvironment.

**Abbreviations**
(H)ERV: (human) endogenous retrovirus
LINE: long interspersed nuclear element
LTR: long terminal repeat
SINE: short interspersed nuclear elements

## Introduction

As it became clear that genetic material was composed of DNA it was relatively simple to estimate the number of possible genes in the human genome by calculating how much DNA was present in a single cell. If the average gene encoded the information for a protein of 100 amino acids, then it contained about 300 nucleotides while the genome contained approximately three billion base pairs, or the capacity for about 10 million genes. There were many assumptions in this calculation, so it was considered too high a number. This problem came to be called the *c*‐value paradox. Why was there so much DNA in each cell?

The first clue that the sequence structure of the genome was more complex came from following the rate of re‐association of DNA double strands (a second‐order rate equation) and the clear finding that genomes contained repetitive sequence elements found at various concentrations, totaling up to 25–65% of eukaryotic DNA 1. It was immediately proposed that such sequences played a role in shaping gene regulation 2. The nature of these repetitive DNA sequences became clear upon the sequencing of the human genome 3, 4. About 50% of the human genome is composed of repetitive sequence elements whose origins derive from several diverse biological processes (Table 1).

**Table 1:** Classes of repetitive elements in the human genome

Repetitive element	Type	Estimate of number of copies	Percentage of genome (%)	Typical length (bp)
Satellite	Tandem	427,000	3	2–100
SINE element	Interspersed	1,800,000	15	100–300
DNA transposons	Interspersed	465,000	3	200–2,000
LTR transposons	Interspersed	718,000	9	200–6,000
LINE retrotransposon	Interspersed	1,507,000	21	500–8,000
Total		4,917,000	51	

These repeats can be classified into autonomous and non‐autonomous transposition species. Autonomous species have their own active mechanism for replication and include DNA transposons and retrotransposons. DNA transposons employ DNA‐dependent DNA synthesis to replicate a transposon either in the genome or excised from the genome, and transpose it to another site in the genome. By contrast the LINE and the LTR (long terminal repeat) retrotransposons employ reverse transcriptase to copy RNA into DNA, which is inserted into the genome in a quasi‐random fashion. A subset of retrotransposons derive from the LTR‐containing retrotransposons, which originate from exogenous retroviruses that infect germline cells and insert their DNA into the genome, thus becoming endogenous retroviruses (ERVs). These viruses can then amplify their copy number by transcribing their DNA and copying the RNA into DNA that inserts into chromosomes at diverse sites.

Non‐autonomous transposition repeats include the SINE elements (short interspersed nuclear elements), which are thought to arise from RNA species in the cell that are retrotransposed by active retrotransposons, the LINE retrotransposon being the most prevalent. The RNAs copied into SINES are all produced by RNA polymerase III, and the most common SINES derive from tRNAs, 7S RNA, and 5S RNA components of the ribosomes in a cell, i.e. the most abundant RNAs. The copying of mRNA to produce DNA pseudo‐genes adds another small number of repetitive elements to a genome. Tandem satellite sequences are classically thought to arise by stuttering at DNA replication forks and copying the same DNA sequences one or more times. They then can be expanded by unequal crossing over during recombination. More recent data indicates that some centromeric and pericentromeric repeats in human or mouse chromosomes (producing HSATII or GSAT RNAs, respectively) can borrow an endogenous reverse transcriptase, produce a DNA copy, and insert back into the centromeric region from where they originate 5. Together, these repetitive elements have found similar, but distinct mechanisms of expansion in genomes, generating a vast landscape upon which evolution can act. The evolution of p53's function is deeply entangled with this landscape.

## Do repetitive elements have a function selected for by a host?

Most transposable elements in the human genome were inserted into the germ line millions of years ago and as such have accumulated mutations or have been only partially copied by reverse transcription, becoming defective movable elements. Of the approximately 1.5 million LINE elements in the human genome, it is estimated that about 80–100 elements are still fully functional and capable of transposition 6. Of the many human ERVs (HERVs), all are likely partially defective but some of the viruses do make viral particles (HERV‐L and K), possibly by employing the many DNA copies in a genome to produce some functional proteins that complement the defects in each viral genome and assemble a virus particle.

Whether these repetitive elements are selfish genetic parasites in our genome that find a way to self‐replicate, or have a functional role to play in normal or pathological situations remains unclear, but this has been an intensively studied subject, particularly regarding their role in the regulation of gene expression 7. What is quite clear is that cells go to great lengths to silence the genomic information contained in repetitive elements, at least during most normal developmental and adult phases of our lifespans. Viable or movable repetitive elements in the mouse or human genome appear to be able to be transcribed, and insert new DNA copies into the genome at each generation in the germ line during meiosis or early in development in stem cells 8, 9, 10, 11, 12. This could accelerate the rates of evolution and give rise to offspring that could be selected for or against in subsequent generations.

Recently it has been shown that selected LINE1 elements can replicate and move during normal neuronal development producing somatic mosaicism in mouse and human neurons of the central nervous system 13, 14. More recent data using single cell sequencing has revealed that LINE1 retrotransposition does occur in individual human neurons. The frequency of these events may not be as high as previously thought, though their general contribution to diversity is still being understood 15, 16. Expression of HERVs has also been found to be potentially important in normal human development. HERV‐K viral particles have been shown to be expressed at the 8‐cell stage of human embryogenesis, which may lead to species‐ and individual‐specific cellular modulation in early development 17. Intriguingly, this work also suggested that HERV‐K proteins may provide an immunoprotective effect against certain exogenous viruses. In sum, the activation of repetitive elements appears in critical stages of early human development, germ cells, and plastic adult cells providing a mechanism for driving inter‐ and intra‐individual variation as well as potential immunomodulatory effects. Thus it is possible repeats have, in some cases, a beneficial functional effect for the normal host beyond their parasitic behavior.

## The p53 protein functions to restrain the movement of repetitive elements

The p53 protein is a transcription factor whose levels and activity are induced by a wide variety of stresses such as genomic damage 18. This protein is highly conserved with regard to its primary amino acid sequence and protein structure. Furthermore, the DNA sequence specifically bound by this protein is highly conserved among diverse species from sea anemone to humans 19, 20. In the sea anemone, *Drosophila* and *Caenorhabditis* the p53 protein is mostly expressed in the germ line and it enforces fidelity of genome integrity by killing (using a conserved apoptotic mechanism) cells containing DNA damage and abnormal genomes. In vertebrates, the p53 protein is also expressed in somatic tissues and enforces genome stability by acting as a tumor suppressor gene. Strikingly, it is the most frequently mutated gene across human cancers.

Recently Wylie and co‐workers 21 employed both *Drosophila* and zebrafish models to demonstrate that the wild type p53 protein, genetically interacting with piwi RNA protein complexes in the female germ line, prevented the movement of repetitive mobile elements. These piwi RNA protein complexes have previously been shown to be important for repetitive element silencing across species 22, 23, 24. Females with mutant p53 genes had increased levels of mobile element RNAs. As expected for random integration events of DNA copied from these RNAs, the eggs had reduced fertility and defective egg formation the extent of which was variable from animal to animal. It had been previously shown with *Drosophila* that double‐stranded DNA break formation in meiosis, carried out by the highly conserved meiotic spo11 nuclease, increased the p53 protein levels in eggs undergoing recombination 25. Increased p53 levels are found in all eukaryotes examined to date in response to single‐ or double‐stranded breaks in DNA. Wylie and co‐workers demonstrated that the p53‐/spo11‐ double mutants (with fewer DNA breaks in the DNA and no recombination) have much reduced levels of RNAs from mobile elements in their eggs. Thus spo11 is epistatic to p53 for derepression of mobile element transposition. Not only are p53 and spo11 in the same pathway but mutations in the proteins of the piwi RNA protein complex have also been shown to induce or increase p53 levels and activity relating p53 function to the piwi RNA protein complex activity 26. Replacing the defective *Drosophila* p53 gene and protein with a wild‐type human p53 protein‐reduced retrotransposon RNA levels in eggs, while replacing the defective *Drosophila* gene with a mutant human p53 gene retained high levels of retrotransposon RNAs in eggs. In zebrafish with wild‐type p53 levels, the open reading frame −1 (ORF‐1) of a LINE element was silent in the embryo, but high levels of ORF‐1 protein were expressed in p53−/− embryos. In p53 wild‐type embryos, the integrated DNA LINE transcriptional enhancer region was shown to contain the transcriptionally repressive histone chromatin marks, H3K9me3. In zebrafish with a mutant p53 gene, these repressive marks were not found and LINE RNA was produced.

In parallel, it has been known for some time that cancers can express LINE‐1 and other LTR retrotransposons (HERVs), as well as SINES, and a many other repetitive elements 27. Wylie and co‐workers demonstrate a correlation between the expression of LINE‐1 ORF‐1 and the presence of mutant p53 genes in Wilms tumor and colon cancers consistent with their hypothesis that p53, along with DNA methylation, histone modifications, and the piRNA protein complexes regulate repetitive element movement. Their publication makes the compelling case that p53 plays a central role in regulating repetitive elements in germ line and somatic cells 21. A number of previous publications are consistent with this idea, as will be discussed in the next section.

## The mechanisms that permit p53 to restrain repetitive elements

The p53 gene and its protein are a compelling example of the co‐evolution of genome stability and regulation of endogenous elements. Hoh and co‐workers 28 developed the first algorithm to identify the genes regulated by the p53 protein employing the consensus p53 DNA sequence binding sites. It was soon found that p53 binding sites appear in the THE1‐MaLR family of transposons 29. Wang and co‐workers found that p53 binding sites within ERV‐derived LTRs accounted for 30% of p53 binding sites, and that p53 regulated the expression of many nearby genes 30. When this algorithm was employed by Harris and co‐workers 31 they found that LINE‐1 elements contained a 15 nucleotide sequence in their promoter that binds a wild‐type p53 protein. Recent work has found that the p53 response elements associated with transposons are less conserved but of higher occupancy and lower accessibility than non‐transposon associated response elements 32. Moreover, transposable elements have been further linked to non‐coding RNA regulation, where p53 has emerged to have a regulatory role 33, 34.

Most of the p53 protein binding sites in defective LINE‐1 elements are negative regulators of transcription. This negative regulation was also shown to be the case for p53 DNA binding sites found in *cis*‐acting regulators of HERV‐1‐LTRs 35. However, a p53 LINE‐1 retrotransposon DNA binding site that is a recent addition to the primate germ line (20 million years ago) can increase the LINE‐1 RNA transcription (in the absence of CpG methylation, chromatin repression, and piRNA clustering). Based upon these observations, most repetitive DNA sequences are negatively regulated by genome CpG methylation, heterochromatin repression, and piwi RNA protein complexes. When these restraints upon expression are lost the wild type p53 protein can promote transcription of some LINE‐1 genomes, this results in an RNA to DNA transposition and the insertion of this DNA is always mediated by double‐stranded breaks 36. The persistence of double‐stranded breaks, which are now amplified by increased transposition, activates p53 resulting in cellular apoptosis and the enforcement of genomic stability in the organism by the killing of those cells with transpositions, reinforcing the regulatory role. In *Drosophila*, DNA damage by gamma radiation results in germ line p53 activation and the transcription of several genes (reaper and sickle), which initiate apoptosis. Thus, germ line and somatic fidelity are enforced by death.

In the germ line of female mice (but not male mice) with a p53 mutation, litter sizes are smaller, as was the case with *Drosophila* females with p53 mutants 26, 37. In addition, about one third of the female offspring produced from p53−/− X p53−/− crosses had birth defects in the C57Bl/6 genetic background 37. When the insulin‐like growth factor‐2 (IGF‐2) gene CpG methylation pattern was examined in these p53−/− offspring many of these defective mice had an altered epigenetic pattern 38. These data suggest that the absence of the p53 protein could impact the fidelity of CpG methylation of epigenetic marks. Several observations support this hypothesis. Jackson‐Grusby and co‐workers 39 knocked out the DNA methytransferase‐1 gene (Dnmt1) in mouse cells. After two cell divisions in the absence of this methyl‐CpG copy transferase, the cells underwent a p53‐mediated apoptosis indicating that the p53 protein senses and ensures the fidelity of this methylation pattern. Mutant p53 fails to do this, and enhances epigenetic plasticity. Takahashi and Yamanaka 40 had first demonstrated that the addition of four transcription factors (c‐myc, KLF‐4, Oct‐4, and Sox‐2) to fibroblasts in cell culture results in the epigenetic reprogramming of these cells and the induction of pluripotent stem cells (iPS cells). However, if this is done in the absence of wild‐type p53 the efficiency of the formation of iPS cells can increase from 10‐ to 100‐fold, the time it takes to produce iPS cells is shorter by 5‐ to10‐fold and one can eliminate the need for c‐myc and KLF‐4 in producing iPS cells 38, 41. Clearly there is a relationship between p53 surveillance of epigenetic marks and altered marks activating wild‐type p53 42.

**Figure 1:**
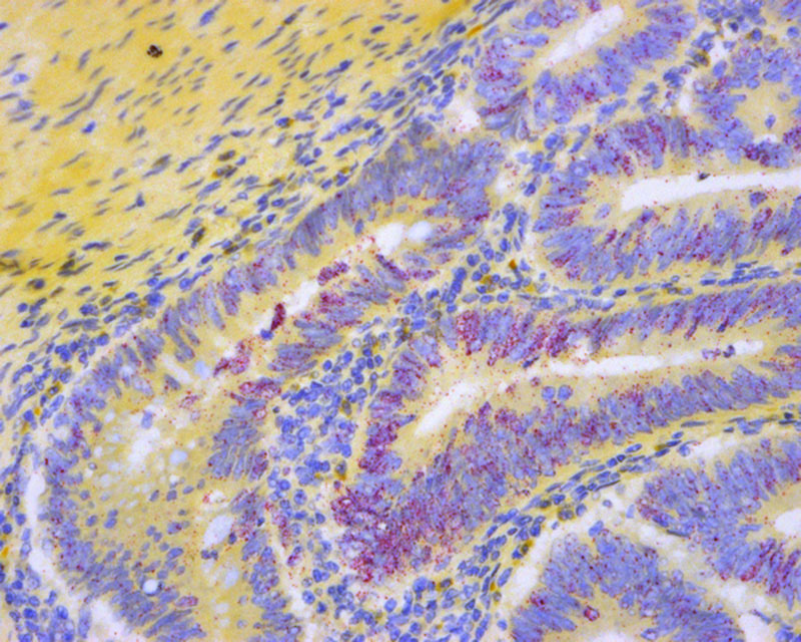
Primary colon adenocarcinoma stained with RNA in situ hybridization assay for HSATII non‐coding RNA (red dots) and counter stained with hematoxylin. The magnification is 200×. Colon cancers are often p53 deficient. Detection shows abundant HSATII expression in tumors.

Indeed the first suggestion that the p53 protein directly regulates the expression of repetitive DNA elements in cancerous cells, was demonstrated by Haoudi et al. 43; Noutsopoulos et al. 44; and by Leonova and co‐workers, who showed that p53 mutations were required to observe the epigenetic changes and the expression of SINE repeats, satellite repeats, and other lncRNAs 45. The treatment of mouse cancerous cells containing a mutant p53, but not wild‐type p53, with 5′aza‐2′‐deoxycytidine (which prevents CpG methylation in DNA) resulted in large increases in the transcription of these repetitive elements in these cells 45. The presence of double‐stranded repetitive RNAs and DNA:RNA complexes induced the innate immune system in these cells to produce interferon, which led to cell death 45. A wide variety of human and mouse carcinomas were found to express abundant satellite and LINE repeats compared to normal tissues 46. The highest levels of cancer‐specific DNA repeats observed were the HSATII and GSAT satellites in human and mouse tumors, respectively (Fig. 1). Interestingly, these same HSATII and GSAT satellites were identified by computational sequence analysis for their unusual sequence motifs (high‐CpG content) compared to other RNA transcripts, and that these motifs could trigger the innate immune system to produce cytokines 47. Similarly, results indicating that HERV expression, can initiate the innate immune response in a variety of malignancies leading to alterations in the local tumor microenvironment that can affect the response to immunotherapies 48, 49, 50. This parallels the suggestion that HERV‐K expression in human embryos have immunoprotective effects 17. Altogether, the suppression of these repeats by p53 appears to be important in modulating the tumor cell innate immune response across many malignancies. However, the relative contribution of these different repeats to the shaping of the immune microenvironment remains to be determined. Thus there is a growing literature that demonstrates a functional consequence of expression of repetitive elements in a cell resulting in its recognition by the immune system.

In addition to these effects on the innate immune system, the expression of repetitive elements in cancer can drive genomic instability through retrotransposon insertional mutagenesis 51, 52, 53 as well as chromosomal instability driven by satellite repeat expression 54, 55. The mutations that drive tumors in p53−/− germline knockout mice result from genomic instability and are most commonly deletions, gene amplifications and rearrangements on a background of aneuploidy and chromotrypsis 56. Based upon all of these observations it is not surprising that the loss of p53 gene functions through mutation is the single most common gene mutation observed in human cancers.

## Conclusions

The first indication of a conserved p53 gene in evolution is found in modern choanoflagellates and sea anemones (early multicellular organisms) which can be traced back to a common ancestor with humans of about 800 million to a billion years ago. Not only is the amino acid sequence and protein structure of p53 conserved but the specific DNA binding site sequences employed by p53 are also conserved over this time frame. In those organisms, p53 monitors the fidelity of the germ line by killing cells with DNA damage. The repetitive DNA elements of transposons initiate double‐stranded DNA breaks during movements in the genome activating the p53 protein and killing cells that undergo transposition. As such the p53 protein and the regulation of genomic instability by transposition have co‐evolved in organisms, eventually moving the p53 gene functions from the germ line into the somatic tissues of vertebrates where it becomes a tumor suppressor gene as well as a germ line fidelity factor. During this evolutionary process some transposable elements have acquired p53 DNA binding sites. After a double‐stranded break, p53 is activated for transcription and some of the p53 DNA binding sites in the LINE elements attract the p53 protein and start a new round of mRNA synthesis and retrotransposition, setting up a positive feedback loop and resulting in p53‐mediated cell death. When a germ line cell escapes death the newly inserted retrotransposon DNA may now activate adjacent genes for p53‐regulated transcription, impacting the p53 signal transduction pathway which appears to have evolved in this manner.

Three biological processes play a role in regulating the expression and movement of transposons in the genome. The first is the methylation of cytosine residues in DNA at CpG sites and the associated chromatin modifications that produce condensed heterochromatin preventing access to enhancer/promotor elements required for transcription. The second process employs piwi proteins and their associated piwi RNA complexes, which prevent transcription of repetitive elements and can even degrade retrotransposon RNAs after their transcription. The third process that triggers a response to double stranded breaks in the genomic DNA for recombination and the insertion of retrotransposons is the p53 stress response which can initiate a program of transcription resulting in cell cycle arrest, senescence, or apoptosis eventually repairing the DNA damage or killing the cell. The loss of any one of these three processes can lead to movement of transposons and even genomic instability. Indeed these three processes communicate with each other and are interactive. Mutation of piwi proteins increases and activates the p53 transcription factor 21. The alteration or failure of copying methylated CpG residues by DNA methyl‐transferase results in the activation of p53 transcription and the killing of the cell with an altered pattern of methylation 39. A mutant p53 protein alters the probability of faithfully copying methylated cytosine residues in genomic DNA and also permits the removal of cytosine methylation from CpG residues during the reprograming of cells to produce stem cells 38. Clearly these three biological processes are cooperative and interactive with loss of CpG methylation or piwi protein mutations activating the p53 transcription factor to kill the cell. Thus the p53 protein acts as a suppressor of changes in CpG methylation and piwi mutational alterations.

The detailed mechanisms that mediate this communication between these three processes remain unclear. The epigenetic reprogramming of cells play an important role in the origins and evolution of cancers as do mutations in the p53 gene, which is the single most common mutation observed in human cancers. Both of these changes in cells can lead to trans‐determination of cell types and an epithelial‐mesenchymal transition observed in cancers. There is not a great deal of information about a role for the piwi protein‐RNA complexes in cancers but it seems a natural direction to explore. The movement of retrotransposons in cancers results in insertions, double‐stranded breaks in the DNA and new transcriptional programs all of which increase the rates of cellular evolution and possibly drug resistance. The transposition of a DNA element in a single cell now identifies that cell as unique (in its genome and possibly its gene expression) in an organ. The expression of retrotransposon RNA, DNA, and proteins should have an impact upon the responses of the immune system in recognizing cancers 6, 47, 57 or alerting the immune response. These ideas bring up many new avenues to explore in the future.
